# Alteration in emergency theatre prioritisation does not alter outcome for acute appendicitis: comparative cohort study

**DOI:** 10.1186/1749-7922-4-22

**Published:** 2009-06-08

**Authors:** Stefano Partelli, Sabina Beg, Juliette Brown, Soumil Vyas, Hemant M Kocher

**Affiliations:** 1 Barts and the London HPB Centre, The Royal London Hospital, Whitechapel, London E1 1BB, UK

## Abstract

**Background:**

Despite dedicated emergency theatre, emergency surgery can be often delayed due to competing urgencies, suggesting a need for innovative theatre time management.

**Aim:**

To investigate if a change in the emergency theatre prioritisation affects outcomes for a common urgent operation such as appendicectomy.

**Methods:**

We prospectively recorded data from 67 patients undergoing appendicectomy, for two cohorts of patients: before and after change in theatre prioritisation: Group 1 (Jan-Mar) and 2 (Aug-Oct) respectively. Demographic and peri-operative data, time from admission to surgery, postoperative length of stay and total length of stay and complications were compared.

**Results:**

The two groups were comparable with regards to gender, age, time of admission and histological confirmation of appendicitis. No differences between the two groups were found regarding time from admission to surgery (24.4 (95% CI 11.2;27.6) hours versus 16.1 (95% CI 10.4;21.7) hours, Mann-Whitney U test, p = 0.35), postoperative length of stay (90.8 (95% CI 61.4;120.1) hours versus 70 (95% CI 48.3;91.6) hours, Mann-Whitney U test, p = 0.25) and total length of stay (115.2 (95% CI 84.6;145.7) hours versus 86 (95% CI 61.6;110.4) hours, Mann-Whitney U test, p = 0.07) as well as complication or re-admission rates.

**Conclusion:**

A change in the emergency theatre prioritisation does not affect outcome for appendicectomy. Provision of a second emergency theatre could be a solution to reduce the delays in acute surgical operations.

## Background

Appendicectomy is amongst the commonest acute surgical operation of intermediate nature, which if not treated in a timely manner could be life-threatening. During the working week (Monday–Friday) urgent and emergency surgery is often delayed until the elective operating list has finished [1], particularly for non-life or limb threatening situations. The American College of Surgeons recommend the provision of a dedicated trauma operating theatre [2];this intervention could reduce the incidence of complications [3]. In the UK, the National Confidential Enquiry into Patient Outcome and Death (NCEPOD) annually recommends changes in management policies affecting patient outcomes based on national audits. In 1992 NCEPOD recommended the provision of dedicated emergency theatres in the UK[4]. Several authors have reported improvement in the quality of emergency services by providing easy access to theatres during daytime and effectively minimising out-of-hours operating [5-9]. Apart from these two instances, we could not uncover any other national audit or guidelines.

Nevertheless NCEPOD report in 2003 suggested that only 58% of all NHS hospitals (in the UK), had a designated theatre for emergency surgery during daytime [10]. Furthermore, even the presence of a single dedicated emergency operating theatre may not be sufficient for a tertiary referral centre, catering to a diverse, socio-economically deprived population and offering specialist trauma surgical services (which takes precedence over most other urgent surgical procedures) [11]. We have previously shown that precisely for this particular reason, common operations such as abscess drainage and appendicectomy stay longer in hospital [11].

We, therefore, convinced the hospital management for a change in emergency theatre utilisation. In the absence of additional space for another parallel day-time emergency theatre, the hospital management implemented a change in emergency theatre prioritisation. Hence we audited whether such a change affected outcomes for appendicectomy.

## Methods

For the purpose of this study, in order to obtain two comparable homogenous groups we prospectively collected anonymous data over two time periods: January–March 2008 (Group 1) and August–October 2008 (Group 2). The intervening period (April 2008 – July 2008), was the transition period whilst the below mentioned changes were implemented but were inconsistent with allocation; therefore this period was not analysed. All patients admitted at the Royal London Hospital (RLH) with suspected acute appendicitis were included. Demographic, operative and post-operative details were obtained; time of admission, time of operation, and time of discharge were prospectively recorded.

Before April 2008, the dedicated emergency operating theatres at the RLH worked on "first come first serve" policy, with the flexibility of allowing for immediate surgery, at the clinical discretion of the surgeons and anaesthetists concerned. After April 2008, the dedicated emergency theatre was divided in 3 sessions of 3.5 hours each (divided between 0800 hours to 1830 hours for the five working days), with sessions being systematically allocated to each surgical sub-speciality (General Surgery, Orthopaedics and Trauma, Vascular/Trauma Surgery, Neurosurgery, Renal Surgery, Gynaecologic Surgery, Plastic Surgery and Oral and Maxillofacial Surgery) on different days of each working week. Additionally, an "open session" allowed for any unscheduled emergency operating.

### Statistical analysis

Distribution of continuous variables are reported as median and interquartile range (IQR) (25^th^; 75^th ^centiles). Categorical variables are presented as numbers and percentages. The comparison between subgroups was carried out using Student's t test, or Mann-Whitney U test, (for continuous variables). Qualitative data were compared by the Chi square test or Fisher's exact test when necessary. Statistical analyses were performed in SPSS 16.0 for Windows software (SPSS Inc, Chicago, Illinois, USA). For all comparisons, a two-sided p < 0.05 was considered statistically significant.

## Results

**Figure 1:**
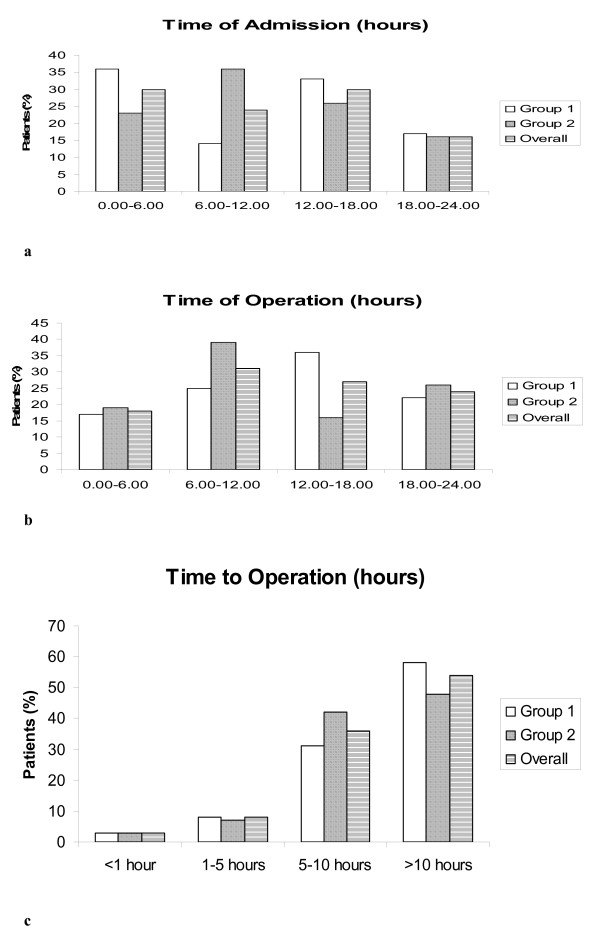
**Distribution of patients admitted, with a suspected diagnosis of appendicitis, during the day clustered by time of admission (a), time of operation (b) and delay from making to diagnosis to operation (c) across both groups and overall**.

Demographic and clinical details are summarized in table 1 with no differences between groups. For the entire cohort of 67 patients the distribution of time of admission (figure 1a), the distribution of time of surgery (figure 1b), showed no difference, allowing us to compare two groups for any delays to theatre. Figure 1c demonstrates time required from decision to operate to time for surgery, again demonstrating no difference (Mann-Whitney U test, p = 0.349). A comparison using mean and 95% confidence interval suggested absence of type II error, though, of course, this cannot be entirely ruled out. Thus no differences between the two groups were found regarding time from admission to surgery (24.4 (95% CI 11.2;27.6) hours versus 16.1 (95% CI 10.4;21.7) hours, Mann-Whitney U test, p = 0.35), postoperative length of stay (90.8 (95% CI 61.4;120.1) hours versus 70 (95% CI 48.3;91.6) hours, Mann-Whitney U test, p = 0.25) and total length of stay (115.2 (95% CI 84.6;145.7) hours versus 86 (95% CI 61.6;110.4) hours, Mann-Whitney U test, p = 0.07).

**Table 1:** Demographic and clinical details

	**Group 1**	**Group 2**		
Period	January–March	August–October	*p*	Test

Number of patients (*n*)	36	31	-	

Males (*n*)	27	17	0.08	Fisher's exact

Age (mean;95% CI)	20.7 (16.6;24.7)	25 (19;31)	0.36	Mann-Whitney U

Perioperative antibiotics (*n*)	15	15	0.63	Fisher's exact

Complications (*n*)	4	0	0.12	Fisher's exact

Confirmed appendicitis	33	28	1	Fisher's exact

Appendix histology*				
Normal	3	4		
Inflammed	19	20	0.07	Fisher's exact
Necrosed	11	2		
Perforated	3	5		

**Figure 2:**
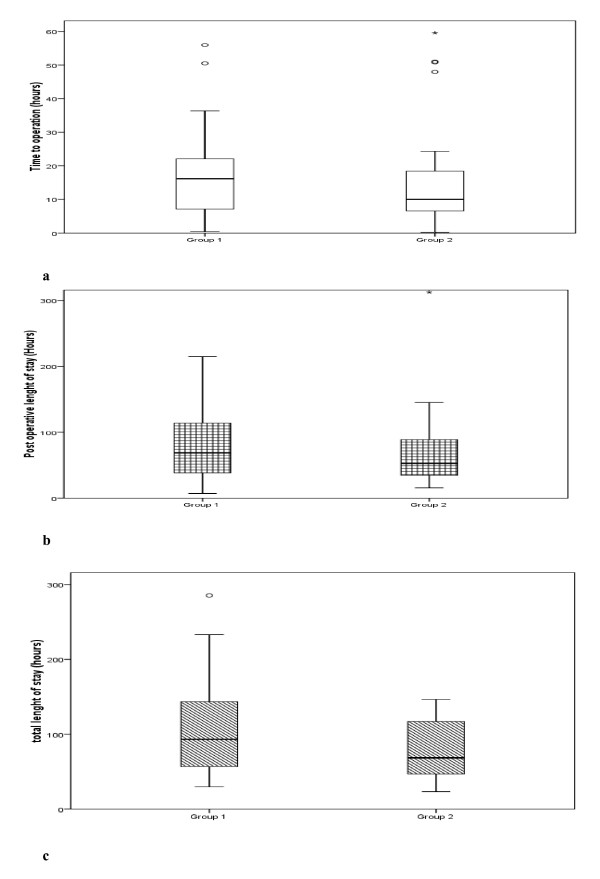
**Comparison of time from admission to surgery (a), postoperative length of stay (b) and total length of stay (c) between the two groups**. Box and whisker graphs represent median ± inter-quartile range.

Four patients had post-operative complications: 3 of these were operated within 5–10 hours from admission while the remaining one was operated 18 hours after the admission. In all the 4 patients requiring readmission within a week of discharge, the appendicectomy was performed with a delay of more than 10 hours. Table 1 summarises the final histological examination with a trend to more complicated appendicitis in group 1(Fisher's exact test, p = 0.07). Figure 2c demonstrates that there was no difference in the overall length of stay (Mann-Whitney U test, p = 0.072), duration of delay to surgery (Mann-Whitney U test, p = 0.35) and length of postoperative stay in hospital (Mann-Whitney U test, p = 0.25).

## Discussion

Our audit in a comparable cohort of patients over two different time periods, after a change in theatre prioritisation policy, did not demonstrate any significant differences in the outcome after appendicectomy. The intention of implementing this change was to effectively reduce waiting times to emergency surgery and hence length of hospital stay – but clearly the present study has failed to demonstrate this effect. There could be numerous reasons for this finding.

Foremost, this could be due to the small sample size, which will require a multi-centre study. Such a study could be hampered by non-homogeneity of the profile of emergency workload. Our hospital is one of the premier trauma units in the UK and the only site of the only Helicopter emergency medical service (HEMS) in London. Despite this, numerically at least emergency general surgery accounts for 64.2% of all the emergency surgical workload with abscesses and acute appendicitis being the two most frequent reasons for requiring theatre [11]. Of course, trauma as well as vascular operations, because of the complexity of pre-operative and operative work and multiple team involvement, take longer duration and therefore occupy a prominent part of the emergency theatre schedule.

Some authors have suggested an increase in post-appendicectomy complications and longer hospital stay associated to the delay to surgery [12,13], whilst others have failed to demonstrate this trend [14-17]; although, of course most patients would prefer immediate surgical procedure [18]. In our cohort only four patients had a complication; of those, three were operated within 10 hours from admission and only one after 18 hours. Our data doesn't demonstrate significant changes in outcome after the appendicectomy, despite changes in theatre prioritisation. The median length of hospital stay was 76 hours, comparable to other publications [13,14].

Delay to surgery is associated with an increased incidence of complications and length of hospital stay after appendicectomy [12,13,19]. Analyzing a large series of 1081 patients, Ditillo et al[12] from the Yale University, USA demonstrated that in adult patients with acute appendicitis, the risk of developing advanced pathology and postoperative complications increases with time; particularly, those risks rise proportional to delay. These results were corroborated by another study from the Wellington Hospital, New Zealand[13] reporting a significant increase in the rate of complicated appendicitis and morbidity when time to surgery exceeded 24 hours. Furthermore, a study from the Massachusetts General Hospital Von Titte et al[19] reported a incidence of perforation of nearly 90% among 40 patients who had a delay of 72 hours or more after the onset of symptoms. On the other hand others have failed to demonstrate this trend [14-17]. Stahlfeld et al. [15] found no difference in operative time, length of stay, wound infections and antibiotic use in patients operated less than 10 hours from the admission. Similar results were shown by Abou-Nukta et al [14] in a cohort of 309 patients when the delays was 12 to 24 hours. Therefore it seems that a short delay (12–24 hours) to surgery does not significantly alter the outcomes after appendicectomies. However, a greater delay (more than 24 hours) can increase the rate of complications. Delay in carrying out appendicectomy may be due to failure to diagnose the condition accurately, thus resulting in higher incidence of complicated appendicitis (necrosis or perforation) [20]. Over a 25 year period, with increasing use of CT scan and laparoscopy, however there has not been any associated decrease in rate of perforated appendicitis[21]. In our first cohort (group 1), there was a trend towards a delay of mean of 24 hours which may explain a trend towards more complicated appendicitis (table 1).

The median time from admission to operation, the median postoperative and total length of hospital stay were minimally reduced after the changing the theatre prioritisation scheme but these results failed to reach a statistical significance. Utilization of the operating theatre (OT) should not only to guarantee that the greatest number of cases are done, but also consider the costs involved [22]. When additional OT capacity is available, it should be planned with multiple variables in mind such as sub-specialities with the greatest contribution margin per OT hour, as well as those that have minimal need for limited resources such as intensive care unit beds[23]. Mainly due to financial circumstances it is difficult to provide one or more dedicated emergency OTs even if it is strongly desired based on clinical needs [24]. Day case surgery can be severely affected by the increase of emergency admissions. *Nasr et al *reported that 40% of all planned elective surgical operations were cancelled, mainly due to bed unavailability because of the overflow of emergency admissions [25]. Robb et al confirmed the increasing role of the bed unavailability in the cancellation of elective surgical cases and additionally demonstrated cost implications[26]. Vinukondaya et al reported that emergency surgery during the operating list is the reason for cancellation of elective surgery in the 13.9% of the cases [27].

In other countries the main cause for emergency surgery delays is not due to the absence of a dedicated emergency OT. Data from 498 patients form the University College Hospital of Ibadan, Nigeria, over a three-month period showed that only in 38% of cases booked for an emergency operation, surgery was carried out. The main reason for cancellation was surgeon's unavailability [28]. Changing the operating theatre policy, as demonstrated in this article, allows surgeons to designate and inform the patient more accurately the time of his/her operation. However, it did not necessarily reduce the waiting times to surgery. We feel that provision of a second emergency theatre at all times would be an effective solution to this problem. Patients would be operated upon promptly. This would reduce waiting times to surgery and facilitate quicker discharges from hospital, thereby increasing turnover. This would also be satisfactory for the patients; bed management for the elective patients, thereby increasing volumes of elective work load and shortening waiting list times. The increased costs involved in running the second additional theatres should be balanced against the cost of reduced length of hospital stay. Taking an example from emergency laparoscopic cholecystectomy versus elective cholecystectomy after conservative management, the increased immediate operative cost is neutralized by the reduced length of stay and quicker return to work [29]. More detailed cost – benefit analysis involving multiple hospitals and larger number of patients would be required to lend creditable evidence to support this belief.

## Competing interests

The authors declare that they have no competing interests.

## Authors' contributions

SP, SB, JB, and SV collected data under supervision of HMK. HMK initiated the project; did the analysis and wrote the paper with SP. HMK will act as a guarantor for the manuscript.
